# The Impact of Online Environmental Platform Services on Users’ Green Consumption Behaviors

**DOI:** 10.3390/ijerph19138009

**Published:** 2022-06-30

**Authors:** Yuan Ma, Changshan Liu

**Affiliations:** College of Economics and Management, Shandong University of Science and Technology, Qingdao 266590, China; 17860777312@163.com

**Keywords:** online environmental platform services, environmental attitude, price sensitivity, green consumption behaviors

## Abstract

With the continuous prominence of environmental problems, some online environmental platforms have been built in China. Such platforms provide an important carrier for public to learn environmental knowledge and participate in environmental protection. However, whether such platforms can play a substantive role in promoting users’ green consumption behaviors is still unclear. Focusing on this question, the influence of online environmental platform services on public green consumption behaviors is explored. A model based on the theory of stimulus–organism–response is established to analyze the influential mechanism, using the online environmental platform services as the independent variable, users’ green consumption behaviors as the dependent variable, environmental attitude as the mediator, and users’ price sensitivity as the moderator. Survey data are used to test the model. The empirical results show that online environmental platform services have a significant positive impact on users’ green consumption behaviors. Environmental attitude plays a partial mediating role and price sensitivity negatively moderates the mediating role of environmental attitude. Suggestions are given from the perspectives of platform operators and government. This paper provides both theoretical and practical implications for sustainable consumption.

## 1. Introduction

Environmental problems, such as environmental pollution, resource shortage, greenhouse gases, and haze weather, need to be solved urgently. Public green consumption behaviors (GCB) are indispensable to promoting sustainable development. Although China has effectively promoted the public GCB by issuing relevant policies and carrying out public education, on the whole, GCB are still relatively scattered and small-scale. How to promote the public GCB is currently the main problem. With the development of the new generation information technology and the 5th generation mobile networks, mobile phones have become indispensable tools for the public. Some traditional services have been moved online. To be specific, an online environmental platform called “Ant Forest” was launched by “Alipay” (the world’s leading independent third-party payment operator, a subsidiary of Ali). Many green activities are offered to the public by this platform, such as green travel, recyclable express packaging, etc. Similar online environmental platforms include “Castle Peak School” and “Flying Ant” in China. These platforms, characterized by convenience, interactivity, and sociability, have attracted more and more users. According to the company’s data, the number of users participating in the “Ant Forest” has reached 600 million by 2021, involving individuals of all ages. Behind the upsurge, we have to think: Can such online environmental platforms encourage users to practice GCB? It is very meaningful to analyze the impact of such platforms on the psychology and behaviors of users. 

Extant research shows that the main influential factors of GCB are demographic characteristics, such as age [1] and education level [2]; psychological factors, such as environmental concern [3], moral identity [4], and environmental awareness [5]; and external factors, such as living environment [6], information intervention [7], and social media [8]. In recent times, research from the perspective of online environmental platforms has begun to appear. The effect of a single feature of these platforms on users’ GCB, such as interactive characteristic and gaming features, has been studied [9,10]. In summary, the studies focusing on GCB are still based on the traditional lifestyle and the research considering characteristics of the Internet era is still lacking.

Extant research has confirmed that related services provided by online platforms can affect users’ psychology and behaviors [11,12,13,14]. In this paper, we posit that online environmental platform services (OEPS) will impact users’ GCB. For example, environmental information can enrich users’ knowledge and improve their perception of environmental problems [15]. The communication between platform users will deepen their empathy for environmental pollution and awareness of the contribution of the environmental behaviors they adopt [16]. 

In order to explain the influential mechanism, the stimulus–organism–response theory is adopted. OEPS could deepen users’ understanding of environmental issues and promote their environmental attitude (EA), which can be regarded as an external stimulus. EA, belonging to organism, can make individuals feel their environmental responsibility and encourage their responses [17]. Since the price of green products is always higher than that of general ones, scholars believe that it is an important factor in predicting GCB [18,19]. Consumers’ price sensitivity (PS) is directly related to product price, which reflects the individual’s acceptance of price differences or fluctuations [20,21,22,23]. Therefore, PS was selected as a moderating variable to moderate the relationship between EA and GCB. 

In summary, this paper establishes a research model with OEPS as the independent variable, EA as the mediator variable, the user’s GCB as the dependent variable, and PS as the moderator variable. Hierarchical regression analysis and the bootstrap method were used to test the hypotheses mentioned in the model. Hierarchical regression analysis is often used to test the relationships between variables in empirical research [3,6]. It has the merits of simple operation and easy interpretation. The bootstrap method is suitable for testing complex models, especially models including a mediation effect and moderating effect [24]. Moreover, the bootstrap method can verify the results of hierarchical regression analysis, thus ensuring the robustness of the research results.

Five hundred and seventeen valid questionnaires collected in China were used as research samples. As is known to all, China is the country with the largest population in the world. Therefore, it is helpful to carry out an investigation in China to promote Chinese citizens’ GCB. It can also provide guidance for other countries. 

The potential contributions of this article are two-fold: (1) As an external factor, OEPS could have a direct impact on users’ psychology and behavior. In order to explore the effect of OEPS in-depth, they are divided into three dimensions: enjoyable services, practical services, and symbolic services. (2) In order to explain the influential mechanism of OEPS on users’ GCB, the mediating effect of EA and the moderating effect of PS are analyzed. We hope this comprehensive analysis will provide theoretical and practical implications to stimulate green consumption.

This paper is composed of six sections. After the introduction, Section 2 introduces the stimulus–organism–response theory and develops hypotheses. Section 3 provides the methods. Section 4 provides results, and Section 5 provides discussions and implications. Section 6 presents the conclusions.

## 2. Theoretical Basis and Hypotheses

### 2.1. Theoretical Basis

The stimulus–organism–response (S-O-R) theory proposed by Mehrabian and Russell (1974) holds that external factors affect an individual’s emotional state and emotional perception, and further affect an individual’s behaviors [25]. Stimulation is an external influence that provokes the individual’s response. After being stimulated, the individual will respond consciously or unconsciously, resulting in specific behavior, such as proximity or avoidance of a particular object [26]. The S-O-R theory plays an important role in analyzing the influence of external environment on individuals’ behaviors and has been widely used. For example, Konuk analyzed consumers’ decision-making process in buying green organic food [27] and Tang et al. discussed the impacts of environmental stimulation and internal psychological state on employees’ energy-saving intentions [28].

We posit that S-O-R theory is applicable to the study of the impact of online environmental platforms on users’ GCB. The platforms have a subtle impact on users’ psychology and behaviors by providing specific services, which are external stimulations to users. EA is the psychological change triggered by external stimulations at the organic level. At the same time, users’ GCB are the responses generated by the stimulations of OEPS.

### 2.2. Hypotheses

#### 2.2.1. The Relationship between Online Environmental Platform Services and Users’ Green Consumption Behavior

GCB refers to the consumption behavior that consumers strive to protect the ecological environment and minimize the negative impact on the environment during the purchase, usage, and disposing of goods [29]. Online environmental platforms provide services to their users through information technology and advocate a culture of public welfare participation, which form a novel environmental protection model with the characteristics of convenience, accessibility, intelligence, efficiency, and transparency [30]. As a result, online environmental platforms are regarded as important methods of promoting GCB.

The services of these platforms are diverse, including online environmental protection participation, environmental knowledge learning, environmental information communication and sharing, and the recommendation of environmental protection activities [31]. From the perspective of users’ experience, these services can be divided into enjoyable services, practical services, and symbolic services [32]. Enjoyable services refer to those services with environmental themes that can promote users’ positive emotions or sensory enjoyment [33], such as games, challenging tasks, and other fun activities. Practical services are those foundation services that provide opportunities and channels for users to engage in green consumption, such as old clothing donations, idle items sharing, etc. Symbolic services include such services as participation score ranking and achievement certification, which help users improve self-image, gain social identity, and the sense of group belonging.

Enjoyable services can attract users and increase users’ stickiness [34]. On the one hand, enjoyable services provided by online environmental platforms can enhance users’ experience by providing fun games. On the other hand, users are instilled with knowledge about protecting the environment and saving resources through games and interesting activities. In this way, users’ understanding of green consumption has been improved. Practical services can provide a carrier for users’ green consumption, which stimulate and facilitate users’ GCB [35]. Symbolic services in platforms give users the opportunity to exhibit their achievements. Users who actively participate in environmental protection activities in the platform can receive awards from the platform. These awards include active credits, honor certificates, etc. In order to obtain the corresponding awards or unique honors, users must deliberately change their behavior [36] and implement GCB in real life. For example, in order to obtain “energy value”, a symbol of green consumption, users of “Ant Forest” deliberately choose green travel tools and avoid using disposable tableware. Therefore, the following hypothesis is proposed:

**Hypothesis** **1** **(H1).** *OEPS have positive impacts on users’ GCB*.

#### 2.2.2. The Mediating Role of Environmental Attitude

EA is an individual’s tendency to protect the environment and can be reflected by the person’s standpoint or view on a specific and detailed environmental problem [37]. On the one hand, the environmental information and activities provided by the platforms can deepen users’ understanding and perception of environmental problems [38] and encourage users to establish a correct EA. On the other hand, the negative news about environmental pollution and resource shortage posted by these platforms can arouse users’ emotional resonance, which could make users feel anxious and nervous [39]. With such anxiety and tension accumulating, users will feel the urgency of environmental protection [40]. The sense of urgency could promote users to generate a positive EA. Therefore, we propose the following hypothesis:

**Hypothesis** **2** **(H2).** *OEPS have a positive effect on users’ EA*.

The theory of planned behavior holds that an individual’s behavior does not occur in a vacuum [41]. Attitude is an important precondition that determines behavior [42,43]. Attitude will affect an individual’s conscious response, regulate the individual’s views on specific problems, and, finally, influence the individual’s actual behaviors. EA is the specific attitude towards environmental problems, which helps people to behave pro-environmentally in real life [44]. In addition, a positive EA increases individuals’ concerns about environmental pollution and resource shortage, foster individuals’ enthusiasm to solve environmental problems, and stimulate them to participate in environmental protection behavior [45]. Therefore, EA has a positive effect on users’ GCB. The following hypothesis is posited:

**Hypothesis** **3** **(H3).** *EA has a positive effect on users’ GCB*.

Based on the above analysis, this article posits that OEPS positively affect users’ EA, and EA plays a positive role in generating GCB. Therefore, EA is a mediator in the relationship between OEPS and users’ GCB. Hence, the following hypothesis is proposed:

**Hypothesis** **4** **(H4).** *EA plays a mediating role in the relationship between OEPS and users’ GCB*.

#### 2.2.3. The Moderating Effect of Price Sensitivity

PS is the degree of awareness and responsiveness shown by consumers when they detect price differences in products or services [46]. Organic green foods, energy efficient household appliances, and other green products tend to have a higher price than general products due to their own green attributes, such as energy saving, low pollution, and recyclable [47]. It is the price premium that aggravates the dilemma of green consumption, which is easy to understand but difficult to perform, causing the long-existing attitude–behavior gap [48]. The price of green products or services is an important factor affecting users’ GCB. If users’ PS is high, their willingness to purchase green products or services with relatively high prices will be reduced. PS inhibits users’ enthusiasm to implement GCB. Although EA can promote users’ GCB, PS will inhibit the driving effect of EA on GCB. Therefore, we posit the following hypothesis:

**Hypothesis** **5** **(H5).** *PS negatively moderates the relationship between EA and users’ GCB. That is to say, GCB of users with lower price sensitivity will be more likely to be influenced by EA, ceteris paribus*.

According to the previous analysis, we argue that the enjoyable, practical, and symbolic services provided by platforms stimulate users’ EA promote their active GCB in daily life. In addition, combined with the extant research, users with low PS would be loyal [49] and are likely to be influenced by platform services to implement GCB. Therefore, this article argues that high PS inhibits the mediating role of EA in the relationship between OEPS and users’ GCB. Specifically, the services provided by the platforms have an impact on users’ attitude and behavior. Under the stimulation of OEPS, users will generate positive EA. However, if the price of green products or services is high, users with high PS are likely to choose low-price ones [50] and decrease their GCB. As a result, the effect of platforms on promoting users to implement GCB through EA is restrained. Therefore, we propose the following hypothesis:

**Hypothesis** **6** **(H6).** *PS negatively moderates the mediating role of EA in the relationship between OEPS and users’ GCB*.

**Figure 1:**
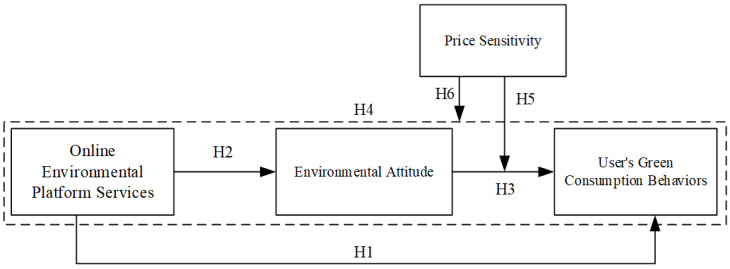
Hypotheses and analytical framework.

The research model developed in this paper is shown in Figure 1.

## 3. Methods

### 3.1. Variable Measurement

Online environmental platform services (OEPS): It is divided into enjoyable services, practical services, and symbolic services from the perspective of users’ experience. The measurement of this variable refers to Mathwick et al. and includes six items [51]. 

Environmental attitude (EA): It is measured from two aspects, the severity of environmental problems and the importance of environmental protection. A total of six items are designed with two reverse items [52].

Price sensitivity (PS): It includes two dimensions: price importance and price search propensity [53]. There are four items in total.

Green consumption behavior (GCB): Four items are used, including clothing, food, housing, and transportation [54].

All variables are scored by a five-point Likert scale. The specific items can be seen in Appendix A.

### 3.2. Sample and Data Collection

Survey data were used. Besides the items of all variables, respondents’ demographic information was included in the questionnaire, such as gender, age, monthly consumption and expenditure, etc. In order to avoid the social desirability bias, some reverse items were included and questions were randomly arranged. The informed consent and some explanations were given to the respondents to eliminate confusion before they filled out the questionnaires.

Considering the disproportion of economy levels in China, the questionnaires were distributed in 3 cities, Qingdao, Shenyang, and Chengdu. The 3 cities belong to East China, Northeast China, and Southwest China. Whether or not they have used online environmental platforms and the reasons for using such platforms were asked before the participants filled out the questionnaires. If respondents answered yes and because of curiosity and other people’s recommendation to use rather than because of concern for the environment, they were invited to participate in the survey. At the same time, this paper reduces the potential impact of other factors on the results in the process of collecting data regarding two aspects. Firstly, the team members told the respondents to try to answer without considering the interference of other conditions. Secondly, for the measurement of green consumption behavior, four aspects of clothing, food, housing, and transportation that are less affected by external factors are selected as the measurement aspects. Initially, 604 questionnaires were obtained. Questionnaires with a too short response time and multiple questions in a row with the same answer were eliminated. Ultimately, 517 valid questionnaires were obtained. The basic information of the 517 respondents is shown in Table 1.

## 4. Results

### 4.1. Reliability and Validity

#### 4.1.1. Reliability

The reliability of the items is analyzed by SPSS25.0 and the results are shown in Table 2. The Cronbach’s alpha coefficients for all variables all exceed 0.8, and the CR values are greater than 0.8, indicating that the combined reliability is good [55].

#### 4.1.2. Validity

Since all the variables were measured with mature scales, the content validity can be guaranteed [56]. The exploratory factor analysis was conducted to ensure the selected scale is applicable. The KMO value is 0.924, and the significance level of Bartlett’s sphericity test is 0.00, indicating that it is suitable for factor analysis. A total of four factors were extracted. The results can be seen in Table 3. Table 2 also show that the AVE value of each variable is greater than 0.5, indicating that the convergent validity is good. The results in Table 4 show that the square root of AVE corresponding to each variable is greater than the correlation coefficient between this variable and other variable, indicating that the scale for each variable has good discriminatory validity [57]. In addition, confirmatory factor analysis is performed using structural equation modeling to test the construct validity. The results are shown in Table 5. The relevant indicators from single-factor model to the four-factor model are more and more ideal, reflecting that the construct validity of the model is good. χ2/df = 2.218, RMSEA = 0.049, CFI, IFI, and TLI all are greater than 0.9 in the four-factor model, which show that the four-factor model has ideal construct validity and is suitable for further analysis [58].

### 4.2. Common Method Bias Test

A method factor was added to the four-factor model to test whether common method bias exists [59]. The result is shown in the five-factor model in Table 5. After adding the method factor, the construct validity test results of the model do not obviously change: The added values of CFI, TLI, and IFI are less than 0.05, and the value of RMSEA is only reduced by 0.005. The results show that the common method bias of the four-factor model is not serious. 

### 4.3. Descriptive Statistics and Correlation Coefficients

The correlation coefficients between the main variables are shown in Table 4. The OEPS (Mean = 4.087, SD = 0.717) is significantly and positively related to EA (Mean = 4.326, SD = 0.538) and GCB (Mean = 4.256, SD = 0.641). There is a significant positive correlation between EA and GCB. Besides, there are also significant correlations between PS (Mean = 3.838, SD = 0.814) and other variables.

### 4.4. Regression Results 

#### 4.4.1. The Test of the Direct Impact of Online Environmental Platform Services

The results of Model 2 in Table 6 reflect the direct effect. In Model 2, OEPS is set as the independent variable and GCB is set as the dependent variable. The results suggest that OEPS have a significant positive effect on users’ GCB (β = 0.569, *p* < 0.001). Therefore, Hypothesis 1 is verified.

#### 4.4.2. The Test of the Mediating Effect of Environmental Attitude

Regression analysis is used to examine the mediating effect of EA. The test results are shown in Model 1, Model 3, and Model 4 in Table 6. 

In Model 1, OEPS is set as the independent variable, and EA is set as the dependent variable. The results show that OEPS have a significant positive effect on EA (β = 0.543, *p* < 0.001). Hypothesis 2 is checked.

In Model 3, EA is set as the independent variable, and GCB is set as the dependent variable. The results indicate that EA has a significant positive effect on users’ GCB (β = 0.568, *p* < 0.001), which supports Hypothesis 3. 

In Model 4, OEPS and EA are set as the independent variables and GCB is set as the dependent variable. The results show that the positive effect of OEPS on users’ GCB is still significant, but the effect is significantly lower than the results in Model 2 (β = 0.370, *p* < 0.001). The change in the coefficient of influence indicates that EA plays a mediating role between OEPS and users’ GCB. Therefore, Hypothesis 4 is checked.

#### 4.4.3. The Test of the Moderating Effect of Price Sensitivity

The two variables of EA and PS are centralized and multiplied to obtain a new variable, named “interaction”. The control variables, independent variable, mediating variable, moderating variable, and interaction are put into Model 5 and Model 6. The results can be seen in Table 6.

In Model 5, EA, PS, and interaction are set as the independent variables, GCB is set as the dependent variable. The results show that interaction has a significant negative impact on users’ GCB (β = −0.130, *p* < 0.001), which verifies that PS plays a negative moderating effect on the relationship between EA and users’ GCB. Therefore, Hypothesis 5 is checked. 

In Model 6, OEPS, EA, PS, and interaction are set as the independent variables and GCB is set as the dependent variable. The results reveal that the interaction has a significant negative impact on GCB (β = −0.094, *p* < 0.05), suggesting that PS negatively moderates the mediating role of EA. Therefore, Hypothesis 6 is checked.

**Figure 2:**
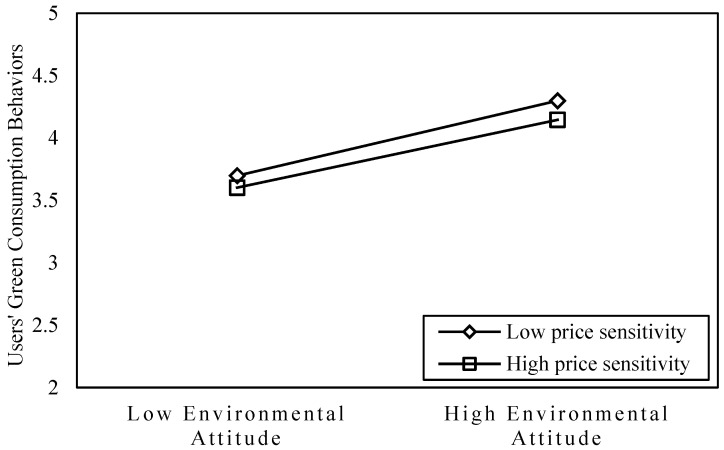
The moderating effect of price sensitivity on the relationship between environmental attitude and green consumption behavior.

In addition, this article uses graphic to visually reflect the moderating effect of PS on EA-GCB relationship, as shown in Figure 2. The results indicate that users with low PS are more motivated to adopt GCB at the same level of EA, showing a negative moderating effect of PS on the relationship between EA and GCB. 

### 4.5. Robustness Test

The bootstrap method is used to test the robustness of the results [60]. The number of samples is 5000 and the confidence interval is 95%. The results can be seen in Table 7, Table 8 and Table 9.

The results in Table 7 show the mediating effect of EA. The total effect value of OEPS on users’ GCB is 0.3303, and the 95% confidence interval is [0.2593, 0.4013], excluding 0. The mediating effect value of EA between OEPS and GCB is 0.1784, and the 95% confidence interval is [0.0931, 0.2846], excluding 0. The results support the direct effect of OEPS and the mediating effect of EA, verifying H1, H2, H3, and H4. 

The results of the moderating effect of PS on EA-GCB relationship are shown in Table 8. The moderating effect value of PS is −0.1242 with the *p* value less than 0.01. The upper and lower limit interval of 95% confidence is [−0.1980, −0.0504], excluding 0. Therefore, PS has a negative moderating effect on the EA-GCB relationship, verifying H5.

The bootstrap method was used to examine the indirect effect of OEPS to users’ GCB under the moderating effect. The method focuses on the mediated effect values and the 95% confidence intervals of users’ EA between OEPS and users’ GCB under three different PS conditions (mean and mean plus or minus one standard deviation). As can be seen from Table 9, changes in users’ PS causes the mediating effect of EA to change. Compared with the mediating effect of EA under low PS, the effect under high PS level is reduced by 34.59%. The results suggest that the mediating role of EA in the relationship between OEPS and GCB becomes weaker as PS level gets higher, which also reflects that PS limits the effect of OEPS in promoting users’ GCB. In other words, it is likely that the high price of green products will have a negative impact on the role of OEPS in promoting users’ GCB, especially for users with high PS. Thus, H6 is further verified.

## 5. Discussions and Implications

### 5.1. Discussions

Only few extant studies demonstrate that some special features of online environmental platforms can affect users’ GCB [9,10]. The research findings of this study strengthen the results of the few studies. The positive effect of OEPS on users’ GCB is corroborated. Specifically, OEPS have a direct effect on GCB. The enjoyable, practical, and symbolic services provided by online environmental platforms can affect users’ psychology and behavior. Users are stimulated by the environmental information and personal achievement feedback. In this case, users generate the urge to practice GCB. Taking “Ant Forest” as an example, in order to obtain the environmental achievements, many users will actively implement GCB in their daily life, such as not using disposable tableware, giving priority to recycled express packaging, etc. This is the true portrayal of the effect of OEPS on users’ GCB. 

Moreover, the information, services, and activities stimulate users to think about environmental issues, encourage them to understand the environmental responsibility that individuals should take, and develop users’ EA. The generated EA will make users pay attention to their own behaviors and promote their GCB. This is consistent with the conclusion that external information or activities can affect users’ attention to environmental issues and then promote users’ GCB [40]. The EA generated by OEPS also becomes stronger with the increase of usage time.

However, users’ enthusiasm to implement GCB is affected by other factors [50]. PS has a negative moderating effect on users’ GCB. OEPS can promote users’ EA and their GCB. When individuals make consumption decisions, they make the most appropriate decisions according to their own conditions. Users with high PS are more concerned about the price of products. When ordinary products can also meet consumers’ needs, they prefer ordinary products with lower prices at the expense of the environment. Therefore, the impact of the price of green products or service and the level of individual PS on GCB cannot be ignored.

### 5.2. Implications

#### 5.2.1. Theoretical Implications

First, the impact of online environmental platforms on users’ GCB was researched. Based on the S-O-R theory, OEPS were taken as the stimulating factors and the services are divided into three dimensions, named enjoyable services, practical services, and symbolic services. The influential mechanism of platform services on users’ GCB was explored in depth. This article expands the research on the factors affecting GCB. The research results can be helpful for scholars and society to realize the importance of the services provided by online environmental platforms to promote users to implement GCB.

Second, this article explained why online environmental platforms can influence users’ consumption behaviors. The results demonstrate that the effect of online environmental platforms on users’ GCB can be achieved through the mediation of users’ EA. EA has been suggested as an important factor influencing individuals’ green consumption intentions and behaviors [61]. This article demonstrated the mediating role of EA in the relationship between OEPS and users’ GCB. It indicated that specific services provided by online platforms would motivate users’ positive EA, then encourage users to practice GCB. This article provided theoretical explanations for existing studies.

Third, the negative moderating effect of PS was examined in this article. PS weakens the role of EA in promoting users’ GCB and restricts the mediating role of EA between OEPS and users’ GCB. Therefore, it has been fully demonstrated that PS would inhibit users from implementing GCB. According to previous studies, PS is a key factor influencing individuals’ green purchase decisions [62]. 

#### 5.2.2. Practical Implications

Firstly, suggestions for platform operators are put forward. First of all, platform operators should strengthen the application of innovative intelligent interaction technology to improve the interaction between users and the platform, as well as the connection between users. In this way, users’ sense of participation and experience will be increased, and users’ enthusiasm to practice GCB can be improved. In addition, it is necessary for platform operators to adjust the acquisition rules of participation points. At present, it is difficult to obtain participation points on some online environmental platforms, which can discourage the enthusiasm of some users and reduce the positive effect of OEPS on users’ GCB. Therefore, platform operators should optimize the rules for obtaining participation points to make users’ green and low-carbon behavior more meaningful and rewarding. At last, platform operators should try their best to meet the diversified interests of users. Specifically, they could consider introducing more incentives and optimizing the symbolic service pattern to meet the spiritual or material needs of different users, thus increasing users’ sense of accomplishment and motivation to participate.

Secondly, suggestions for the government are proposed. The government should strengthen incentives and support for such online environmental platforms, so as to make them more effective. At the same time, product price is an important factor in consumers’ purchasing decisions. The government should strengthen financial subsidies for green products and services and formulate supportive policies for green product manufacturers to reduce the price of green products, thus improving consumers’ acceptance of green products. 

## 6. Conclusions

The following conclusions have been drawn: (1) OEPS have a direct impact on users’ GCB. Enjoyable services, practical services, and symbolic services provide platforms for users, thus improving the enthusiasm of users to implement GCB. (2) The effect of OEPS on users’ GCB is also indirectly realized through EA. Specifically, OEPS encourages users to generate EA. EA can affect users’ understanding of environmental problems and encourage them to implement GCB. (3) PS negatively moderates the relationship between EA and GCB and the mediating effect of EA. For users with high PS, the indirect effect of OEPS is weakened. To sum up, based on the theory of stimulus–organism–response, this paper demonstrates the positive influence of OEPS on users’ GCB. In addition, the results suggest that promoting GCB through OEPS requires the joint efforts of platform operators and government departments.

Some limitations could open new directions for future research. First, the participants of this survey were mainly young and middle-aged, the proportion of elderly participants was small. Therefore, whether the conclusions are suitable for the elderly is still worth exploring. Second, PS was used to measure the acceptability of users to price fluctuation. Although the negative moderating effect of PS has been examined, it is difficult to explain to what extent price fluctuations eliminate the impact of OEPS on users’ GCB, which can be explored in the future. Finally, based on the research findings of this paper, it would also be meaningful to study the actual situation of users’ GCB in real life.

## Figures and Tables

**Table 1:** Respondents’ demographic information.

Variable	Category	Frequency	Percentage (%)
Gender	Man	204	39.50%
	Woman	313	60.50%
Age	20 years old and below	92	17.79%
	21 to 40 years old	258	49.90%
	41 to 60 years old	108	20.89%
	61 years old and above	59	11.42%
Monthly Consumption Expenditure	≤2000 CNY	103	19.92%
	2001–4000 CNY	226	43.71%
	4001–6000 CNY	125	24.18%
	≥6001 CNY	63	12.19%

**Table 2:** The results of reliability and validity test of scale.

Variable	Items	Standard Factor Loadings	Cronbach’s α	AVE	CR
OEPS	OEPS1	0.787	0.962	0.6406	0.9144
	OEPS2	0.826			
	OEPS3	0.807			
	OEPS4	0.813			
	OEPS5	0.794			
	OEPS6	0.774			
EA	EA1	0.772	0.900	0.5298	0.8709
	EA2	0.689			
	EA3	0.695			
	EA4	0.758			
	EA5	0.756			
	EA6	0.692			
PS	PS1	0.627	0.867	0.5365	0.8211
	PS2	0.708			
	PS3	0.786			
	PS4	0.796			
GCB	GCB1	0.752	0.914	0.5194	0.8114
	GCB2	0.711			
	GCB3	0.771			
	GCB4	0.642			

Note: OEPS = online environmental platform services, EA = environmental attitude, PS = price sensitivity, GCB = green consumption behaviors.

**Table 3:** The results of the exploratory factor analysis.

Item	OEPS	EA	PS	GCB
OEPS1	**0.800**	−0.251	0.149	0.121
OEPS2	**0.734**	−0.142	0.225	0.093
OEPS3	**0.809**	−0.256	0.168	0.102
OEPS4	**0.727**	−0.217	0.183	0.122
OEPS5	**0.722**	−0.102	0.231	0.143
OEPS6	**0.713**	−0.226	0.217	0.066
EA1	0.104	**0.743**	−0.079	0.092
EA2	0.213	**0.740**	−0.131	0.070
EA3	0.202	**0.804**	−0.161	0.085
EA4	0.187	**0.787**	−0.177	0.163
EA5	0.141	**0.738**	−0.115	0.128
EA6	0.123	**0.799**	−0.115	0.108
PS1	0.147	0.121	**0.733**	0.113
PS2	0.109	0.212	**0.824**	−0.030
PS3	0.114	0.222	**0.740**	−0.073
PS4	0.103	0.215	**0.751**	−0.071
GCB1	0.109	−0.006	−0.167	**0.801**
GCB2	0.217	−0.053	−0.120	**0.792**
GCB3	0.214	−0.017	−0.138	**0.776**
GCB4	0.134	0.070	−0.102	**0.786**

Note: OEPS = online environmental platform services, EA = environmental attitude, PS = price sensitivity, GCB = green consumption behaviors.

**Table 4:** The AVE square root of variables and correlation coefficient matrix.

	OEPS	EA	PS	GCB
OEPS	**0.641**			
EA	0.537 **	**0.530**		
PS	0.208 **	0.366 **	**0.537**	
GCB	0.564 **	0.565 **	0.309 **	**0.519**
The Square Root of AVE	0.800	0.728	0.732	0.721

Note: ** *p* < 0.01; The data marked in black represent the AVE value corresponding to each variable; OEPS = online environmental platform services, EA = environmental attitude, PS = price sensitivity, GCB = green consumption behaviors.

**Table 5:** The results of the confirmative factor analysis.

Model	Factor	χ^2^	df	χ^2^/df	RMSEA	CFI	IFI	TLI
Single-factor model	OEPS + EA + GCB + PS	1771.840	170	10.423	0.135	0.674	0.675	0.636
Two-factor model	OEPS + EA + GCB, PS	1162.377	169	6.878	0.107	0.798	0.799	0.773
Three-factor model	OEPS + EA, PS, GCB	897.396	167	5.374	0.092	0.851	0.852	0.831
Four-factor model	OEPS, EA, PS, GCB	363.752	164	2.218	0.049	0.959	0.960	0.953
Five-factor model	OEPS, EA, PS, GCB + method factor	289.309	144	2.009	0.044	0.970	0.971	0.961

Note: OEPS = online environmental platform services, EA = environmental attitude, PS = price sensitivity, GCB = green consumption behaviors.

**Table 6:** The results of regression analysis.

Variable	EA	GCB				
	Model 1	Model 2	Model 3	Model 4	Model 5	Model 6
GD	0.100	0.042	−0.028	0.006	−0.040	−0.005
AG	−0.006	−0.038	−0.012	−0.036	−0.018	−0.040
MCE	0.001	0.014	−0.015	0.000	−0.018	−0.001
OEPS	0.543 ***	0.569 ***		0.370 ***		0.357 ***
EA			0.568 ***	0.368 ***	0.469 ***	0.293 ***
PS					0.139 ***	0.129 ***
Interaction					−0.130 ***	−0.094 *
R^2^	0.299	0.321	0.320	0.416	0.347	0.434
ΔR^2^	0.294	0.316	0.315	0.410	0.339	0.426
F	54.662 ***	60.503 ***	60.297 ***	72.690 ***	45.069 ***	55.813 ***

Note: *** *p* < 0.001, * *p* < 0.05; GD = gender, AG = age, MCE= monthly consumption expenditure, OEPS = online environmental platform services, EA = environmental attitude, PS = price sensitivity, GCB = green consumption behaviors.

**Table 7:** The results of the direct effect and the mediating effect using the bootstrap method.

Path	Standard Effect Value	Standard Error	95% Confidence Interval	
			Lower Limit	Upper Limit
OEPS → GCB	0.3303	0.0362	0.2593	0.4013
OEPS → EA → GCB	0.1784	0.0500	0.0931	0.2846

Note: OEPS = online environmental platform services, EA = environmental attitude, GCB = green consumption behaviors.

**Table 8:** The moderating effect of price sensitivity on environmental attitude–green consumption behaviors relationship.

Effect	Coefficient	Standard Error	*p* Value	95% Confidence Interval	
				Lower Limit	Upper Limit
The moderating effect of PS on EA–GCB relationship	−0.1242	0.0376	0.0010	−0.1980	−0.0504

Note: EA = environmental attitude, GCB = green consumption behaviors, PS = price sensitivity.

**Table 9:** The mediating role of EA in the relationship between OEPS and users’ GCB under different levels of PS.

Different Levels of Price Sensitivity	Value	Effect	Boot SE	95% Confidence Interval	
				Lower Limit	Upper Limit
Low PS	3.02	0.1720	0.0441	0.0963	0.2671
Medium PS	3.84	0.1423	0.0447	0.0662	0.2387
High PS	4.65	0.1125	0.0474	0.0299	0.2139

## Data Availability

Data can be available by E-mail if requested.

## Appendix A

**Table A1:** Abbreviation for variable names.

Variable Name	Abbreviation
Gender	GD
Age	AG
Monthly Consumption Expenditure	MCE
Online Environmental Platform Services	OEPS
Environmental Attitude	EA
Price Sensitivity	PS
Green Consumption Behavior	GCB

**Table A2:** The specific items of all variables.

Variable	Item	Source
Online Environmental Platform Service	OEPS1: The online environmental platform has complete functions to meet my green environmental needs	Mathwick et al., 2001 [51]
	OEPS2: The online environmental platform can provide a carrier for my green environmental protection behaviors	
	OEPS3: The online environmental platform provides me with many interesting functions, special activities and friend interaction, which bring me a lot of happiness	
	OEPS4: The online environmental platform provides me with opportunities for game participation and human–computer interaction, which give me a higher sense of experience	
	OEPS5: The online environmental platform provides such functions as achievement presentation, certificate award and so on, which also help me create a friendly image of the environment	
	OEPS6: The activities and services provided by the online environmental platform have a good social image, which also help my participation win good social recognition	
Environmental Attitude	EA1: I pay much attention to the problem of environmental pollution and shortage of resources	Dunlap and Liere, 2008 [52]
	EA2: It isn’t necessary for public to save resources and protect the environment *	
	EA3: Saving resources and protecting the environment are very meaningful for social development	
	EA4: People’s work and life do not make the environmental problems worse *	
	EA5: Waste of resources and environmental pollution are serious environmental problems we are facing	
	EA6: If the current situation continues, we will soon face more serious environmental pollution and resource shortage	
Price Sensitivity	PS1: Price is the most important consideration when I choose to buy a product	Sinha and Batra, 1999 [53]
	PS2: I tend to buy the lowest-priced product when my needs are met	
	PS3: Before making the purchasing decision, I need to collect a lot of information about the price of the product	
	PS4: I think it’s worth to spend time and energy to find a low price	
Green Consumption Behaviors	GCB1: When the economy permits, I prefer to buy recyclable daily products and clothes with reasonable design and green material selection	Wu, 2015 [54]
	GCB2: I would actively purchase organic fruits and vegetables and develop healthy eating habits	
	GCB3: In daily life, I would actively use energy saving appliances	
	GCB4: I would actively travel by public transport	

Note: * Reverse item.
